# Advancing Microbiology through the Discovery of New Microbial Species and Strains

**DOI:** 10.3390/life14050626

**Published:** 2024-05-13

**Authors:** Pabulo Henrique Rampelotto

**Affiliations:** Bioinformatics and Biostatistics Core Facility, Institute of Basic Health Sciences, Federal University of Rio Grande do Sul, Porto Alegre 91501-970, Brazil; prampelotto@hcpa.edu.br

## 1. Introduction

In our pursuit of understanding the intricacies of microbial life, the isolation and characterization of new microbial species and strains play a pivotal role. Recent advances in technology and methodologies have revolutionized the field, enabling scientists to identify and study microbial species and strains with unprecedented precision and efficiency. From innovative culturing techniques to high-throughput sequencing technologies, these advancements have propelled the discovery of new microbes across diverse environments, from human and animal isolates to soil, water, air, and even extreme habitats.

The characterization of new microbial species and strains offers invaluable insights into microbial diversity, ecological interactions, and evolutionary processes. Furthermore, it provides opportunities for exploring novel biotechnological applications, such as bioremediation, biofuel production, and pharmaceutical development, not to mention the key role in the diagnosis and treatment of infectious diseases in humans and animals.

The characterization of new microbial species and strains is a thriving area of research, and the discovery of intriguing new microbes is reported daily. However, the literature on this topic remains fragmented, lacking a concise reference collection that not only reports novel findings but also discusses the methodologies employed in depth and addresses the emerging challenges in the field. To address this gap, a topical collection in the journal *Life* was launched (1 in Appendix A). This thematic collection aims to gather articles that not only present the latest discoveries of new microorganisms but also extensively explore the methodologies and discuss the challenges and future perspectives of this rapidly evolving field.

This initiative seeks to serve as a comprehensive and up-to-date reference for researchers, microbiologists, undergraduate and graduate students, and anyone interested in the advancement of knowledge on microbial diversity and its implications. By dedicating attention to the isolation and characterization of new microbial species and strains, this collection fosters collaboration, knowledge exchange, and scientific innovation within the microbiology community. It serves as a platform for researchers to showcase their findings, discuss methodologies, and address emerging challenges in microbial research.

In this Editorial, I briefly explore the importance of a collection focused on the isolation and characterization of new microbial species and strains, discussing how this thriving area of research contributes to scientific progress and innovation.

## 2. Contribution to Scientific Progress and Innovation

### 2.1. Understanding Microbial Diversity

Microbes play fundamental roles in diverse ecosystems, ranging from soil and water environments to the human body. The discovery of new microbial species and strains provides insights into the vast diversity of microbial life on Earth. Each new species offers unique characteristics, from metabolic pathways to ecological interactions, contributing to the intricate web of life. By characterizing newly discovered microbial species and strains, we gain insights into their ecological roles and the mechanisms driving ecosystem dynamics.

### 2.2. Medical Implications

In medicine, the identification of new microbial species and strains is essential for diagnosing and effectively treating infectious diseases. As antimicrobial resistance continues to escalate, the urgency to find novel antimicrobial agents derived from microorganisms becomes more pronounced. These newly identified microbial species hold immense potential as sources of bioactive compounds with therapeutic properties. Such compounds offer a promising avenue for developing innovative treatments capable of combating drug-resistant pathogens. Furthermore, the exploration of new microbial species and strains not only expands our understanding of microbial diversity but also enhances our ability to develop targeted and effective interventions against infectious diseases. By harnessing the bioactive potential of these microorganisms, it is possible to pave the way for novel antimicrobial therapies that are urgently needed to address the growing threat of antimicrobial resistance in clinical settings.

### 2.3. Veterinary Implications

The isolation and characterization of new microbial species and strains also play a crucial role in animal science and veterinary medicine. Understanding the microbial composition of animals’ microbiomes is essential for maintaining their health and wellbeing. New microbial species and strains may include probiotic bacteria that can enhance gut health, prevent infections, and improve immune function in animals. Furthermore, identifying new pathogenic microbes enables veterinarians to diagnose and treat infectious diseases more effectively, safeguarding animal populations from outbreaks and reducing economic losses.

### 2.4. Environmental Implications

In environmental science, understanding microbial diversity is essential for ecosystem management and conservation. Microbes drive nutrient cycling, soil fertility, and pollutant degradation, influencing ecosystem health. By isolating and characterizing new microbial species, scientists can uncover novel biotechnological applications, such as bioremediation and biofuel production, contributing to sustainable environmental practices.

### 2.5. Technological Innovations

Advances in microbial isolation and characterization techniques propel technological innovations across industries. From biotechnology to pharmaceuticals, new microbial species and strains serve as sources of enzymes, metabolites, and other biomolecules for industrial applications. Harnessing the metabolic potential of microbes leads to the development of novel bioproducts and processes, driving economic growth and innovation.

### 2.6. Scientific Collaboration

A dedicated collection fosters collaboration among researchers, enabling the exchange of information, methodologies, and best practices in microbial isolation, identification, and characterization. This collaborative environment accelerates scientific progress and promotes innovation in microbiology. As such, this collection prioritizes open-access data sharing, ensuring that research findings are accessible to the scientific community worldwide. This transparency promotes collaboration, reproducibility, and data-driven discovery.

## 3. Final Remarks

A collection focused on newly discovered microbial species and strains represents a cornerstone of microbiological research, driving advancements in understanding microbial diversity, function, human and animal health, as well as biotechnological potential. By fostering collaboration, preserving microbial diversity, and facilitating scientific discovery, this collection contributes to the advancement of knowledge and the improvement of environmental, animal, and human health through the lens of the microbial world.

## Appendix A

1. Available online: https://www.mdpi.com/journal/life/topical_collections/ME31S17H04 (accessed on 1 May 2024).

